# Genomic Insights into *Pluralibacter gergoviae* Sheds Light on Emergence of a Multidrug-Resistant Species Circulating between Clinical and Environmental Settings

**DOI:** 10.3390/pathogens12111335

**Published:** 2023-11-09

**Authors:** João Pedro Rueda Furlan, Eliana Guedes Stehling

**Affiliations:** Department of Clinical Analyses, Toxicology and Food Science, School of Pharmaceutical Sciences of Ribeirão Preto, University of São Paulo, Ribeirão Preto 14040-903, Brazil; jpedro.rueda@usp.br

**Keywords:** *Enterobacteriaceae*, genomic analysis, multidrug resistance, plasmid-mediated quinolone resistance, gene transfer, One Health

## Abstract

*Pluralibacter gergoviae* is a member of the *Enterobacteriaceae* family that has been reported sporadically. Although *P. gergoviae* strains exhibiting multidrug-resistant profiles have been identified an in-depth genomic analysis focusing on antimicrobial resistance (AMR) has been lacking, and was therefore performed in this study. Forty-eight *P. gergoviae* strains, isolated from humans, animals, foods, and the environment during 1970–2023, were analyzed. A large number of single-nucleotide polymorphisms were found, indicating a highly diverse population. Whilst *P. gergoviae* strains were found to be circulating at the One Health interface, only human and environmental strains exhibited multidrug resistance genotypes. Sixty-one different antimicrobial resistance genes (ARGs) were identified, highlighting genes encoding mobile colistin resistance, carbapenemases, and extended-spectrum β-lactamases. Worryingly, the co-occurrence of *mcr-9.1*, *bla*_KPC-2_, *bla*_CTX-M-9_, and *bla*_SHV-12_, as well as *mcr-10.1*, *bla*_NDM-5_, and *bla*_SHV-7_, was detected. Plasmid sequences were identified as carrying clinically important ARGs, evidencing IncX3 plasmids harboring *bla*_KPC-2_, *bla*_NDM-5_, or *bla*_SHV-12_ genes. Virulence genotyping underlined *P. gergoviae* as being a low-virulence species. In this regard, *P. gergoviae* is emerging as a new multidrug-resistant species belonging to the *Enterobacteriaceae* family. Therefore, continuous epidemiological genomic surveillance of *P. gergoviae* is required.

## 1. Introduction

Antimicrobial resistance (AMR) is a growing global health concern that affects several sectors. In clinical settings, AMR reduces the effectiveness of antimicrobials and leads to longer hospital stays. Consequently, there is an increase in mortality rates [1]. Furthermore, healthcare-associated infections caused by MDR pathogens are on the rise, which is concerning [2]. In the environment, antimicrobial-resistant strains are circulating especially in water bodies and agricultural soils, impacting human and animal populations [3]. These strains may be reaching food products and leading to difficult-to-treat foodborne infections [4]. In this context, AMR is a multifactorial problem that requires a One Health approach [5].

*Pluralibacter gergoviae*, formerly *Enterobacter gergoviae*, was first described in the 1980s in human clinical specimens and the environment in France and the United States [6,7]. Subsequently, this species was identified as contaminating cosmetic formulations, colonizing the human oral cavity, and causing nosocomial outbreaks [8,9,10,11]. From the 2010s, multidrug-resistant (MDR) *P. gergoviae* strains, harboring clinically important antimicrobial resistance genes (ARGs) and highlighting carbapenemases, began to emerge in clinical settings [11,12,13]. Whilst MDR *P. gergoviae* strains have been described, an in-depth genomic analysis is lacking. Therefore, this study aimed to perform a genomic characterization of *P. gergoviae* strains circulating at the One Health interface and provide genomic insights into AMR. 

## 2. Materials and Methods

### Bacterial Genomes and Genomic Analysis

All *P. gergoviae* genomes (NCBI:txid61647) available at the GenBank^®^ database were downloaded on 1 August 2023. The derived, contaminated, and duplicated genomes were removed. Resistome, virulome, and plasmid replicons were identified using ResFinder v.4.1, VirulenceFinder v.2.0, and PlasmidFinder v.2.1, respectively. Single-nucleotide polymorphism (SNP) counts were determined using CSI Phylogeny v.1.4, with *P. gergoviae* strain FDAARGOS_186 (BioSample: SAMN05004741) as a reference. Antimicrobial susceptibility was predicted using ResFinder v.4.1 and the strains were classified as MDR according to Magiorakos et al. [14]. All aforementioned analyses were performed using tdefault settings at the Center for Genomic Epidemiology (https://www.genomicepidemiology.org/) (accessed on 2 August 2023). The SNP-based phylogenetic tree was visualized and edited using iTOL v.6 (https://itol.embl.de/) (accessed on 10 August 2023). The plasmid contigs were predicted using mlplasmids v.2.1.0 [15]. The insertion sequence elements were identified using ISfinder [16]. The plasmid sequences were manually curated and visualized using Geneious Prime^®^ v.2023.0.4. Comparative plasmid sequence analysis was carried out using BLASTn (https://blast.ncbi.nlm.nih.gov/Blast.cgi) (accessed on 21 August 2023).

## 3. Results

### 3.1. Distribution of P. gergoviae Strains

**Figure 1:**
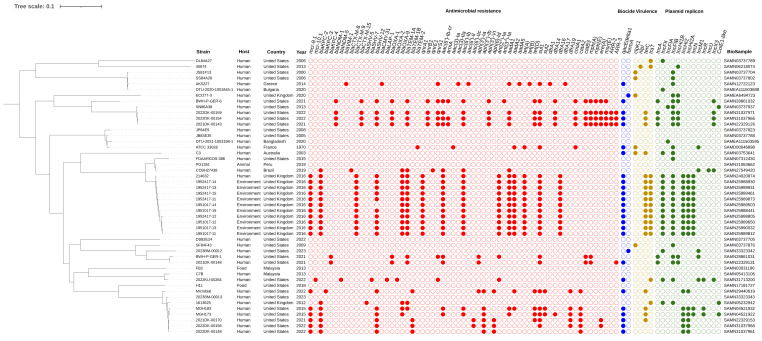
The SNP-based phylogenetic tree of *P. gergoviae* strains distributed worldwide. Strain, host, country, year, and BioSample data were retrieved from the GenBank^®^ database. Red, blue, yellow, and green painted circles represent positive antimicrobial resistance genes, biocide resistance genes, virulence genes, and plasmid replicons, respectively. The tree was rooted at the midpoint.

Forty-eight *P. gergoviae* strains were included in this study. These strains were isolated from humans (abscesses, blood, bronchoalveolar lavage, dentine caries, drainage, endotracheal and tracheal aspirate, feces, gastrojejunostomy tubes, nasopharyngeal swabs, peri-bile duct tissue, right shin wound cultures, tracheostomy sites, and urine), an animal (dog, urine), foods (fish balls and ground beef), or the environment (wastewater), evidencing their circulation at the One Health interface. These strains were obtained from 1970 onwards in countries belonging to the Australian (Australia), American (Brazil, Peru, and the United States), Asian (Bangladesh and Malaysia), and European (Bulgaria, Greece, France, and the United Kingdom) continents (Figure 1).

### 3.2. SNPs Data

SNP differences among all *P. gergoviae* genomes ranged from 27 to 33,993, whereas among human and environmental strains SNP differences ranged from 80 to 33,993 and 27 to 1073, respectively. Between food strains (FB2 and H11), differences of 22,871 SNPs were found. Overall, *P. gergoviae* genomes were grouped according to country or host, but divergences in these characteristics were also observed. The largest clade grouped human and environmental strains (214632, 1951017-11 to -15, and 1952417-11 to -15) from the United Kingdom. Interestingly, human and animal strains (FDAARGOS-386, PG1351, and CCBH27438) from the American continent (Brazil, Peru, and the United States) were clustered. In this regard, the finding of a large number of SNPs indicates a highly diverse population with a range of genetic adaptations that may be related to genetic exchanges or recombination events.

### 3.3. ARG-Producing Strains

A great diversity of ARGs were found to relate to resistance to polymyxins (*mcr-9.1* and *mcr-10.1*), β-lactams (*bla*_KPC-2_, *bla*_KPC-3_, *bla*_KPC-4_, *bla*_NDM-1_, *bla*_NDM-5_, *bla*_VIM-1_, *bla*_CTX-M-8_, *bla*_CTX-M-9_, *bla*_CTX-M-15_, *bla*_SHV-5_, *bla*_SHV-7_, *bla*_SHV-12_, *bla*_CMY-31_, *bla*_LAP-1_, *bla*_OXA-1_, *bla*_OXA-2_, *bla*_OXA-9_, *bla*_TEM-1A_, *bla*_TEM-1B_, and *bla*_TEM-2_), fluoroquinolones [*qnrA1*, *qnrB1*, *qnrE1*, *qnrS1*, and *aac(6*′*)-Ib-cr*], aminoglycosides [*armA*, *aac(3)-Ia*, *aac(3)-IIa*, *aac(6*′*)-Ib*, *aac(6*′*)-Il*, *aac(6*′*)-IIc*, *aph(3*′*)-Ia*, *aph(3*″*)-Ib*, *aph(3*′*)-VI*, *aph(6)-Id*, *ant(2*″*)-Ia*, *ant(3*″*)-Ia*, *aadA1*, *aadA2*, and *aadA5*], tetracyclines [*tet(A)*, *tet(B)*, and *tet(D)*], folate pathway antagonists (*sul1*, *sul2*, *dfrA1*, *dfrA14*, *dfrA16*, *dfrA17*, and *dfrA19*), amphenicols (*cmlA1*, *catA2*, *catB3*), macrolides [*mph(A)*, *mph(E)*, *ere(A)*, and *msr(E)*], and rifamycins (*ARR-2* and *ARR-3*). Some human-derived strains, and all of those from foods and animals, did not contain ARGs. Furthermore, biocide resistance genes were also detected, including *qacEΔ1* (quaternary ammonium compound) in 54.1% of strains and *formA* (aldehyde) in 2023BM-00012 and ECO77-3 strains (Figure 1).

Most strains (58%) carried at least four ARGs. Among them, strains isolated from humans and the environment in Greece, the United Kingdom, and the United States harbored ten or more ARGs related mainly to polymyxins, β-lactams, fluoroquinolones, aminoglycosides, tetracyclines, and folate pathway antagonists. The presence of genes that encode mobile colistin resistance (*mcr-9.1* and *mcr-10.1*), carbapenemases (*bla*_KPC_, *bla*_NDM_, and *bla*_VIM_), extended-spectrum β-lactamases (ESBLs; *bla*_CTX-M_ and *bla*_SHV_), and plasmid-mediated quinolone resistance [PMQR; *qnr* and *aac(6*′*)-Ib-cr*] is highlighted. Interestingly, the coexistence of the aforementioned clinically relevant ARGs was identified in human and environmental strains from the United States and the United Kingdom, respectively. In addition, three human strains (2022DK-00159, 2022DK-00154, and 2021DK-00143) presented more than 20 ARGs (Figure 1). These characteristics contribute to the emergence of multidrug-resistant strains and the spread of clinically important ARGs worldwide.

### 3.4. WGS-Predicted Antimicrobial Susceptibility

Corroborating with the resistome, it was found that most strains (58%) were predicted to be MDR since they presented resistance to one or more antimicrobial agents in three or more antimicrobial categories. Although MDR strains presented resistance to critically important antimicrobials [e.g., polymyxins, penicillins + β-lactamase inhibitors, extended-spectrum cephalosporins (3rd and 4th generations), monobactams, carbapenems, fluoroquinolones, and/or aminoglycosides], they were susceptible mainly to minocycline, tigecycline, and/or fosfomycin. MDR strains were obtained exclusively from humans and the environment, while pan-susceptible strains were recovered from humans, animals, and foods (Table 1).

### 3.5. Plasmid-Mediated Clinically Important ARGs

Various plasmid replicons were detected, including IncA, IncFII, IncFIA, IncFIB, IncHI1B, IncHI2, IncHI2A, IncN, IncM1, IncR, IncU, IncX3, and Col(pHAD28) (Figure 1). Plasmidome analysis revealed that all *mcr*, *bla*, and PMQR genes were housed on plasmid contigs. Furthermore, the association between ARGs and Inc groups was identified in some strains as follows: IncHI2/HI2A plasmid contigs (>140 kb) were identified as harboring the *mcr-9.1* gene embedded on IS*903B*-*mcr-9.1*-IS*1*-ΔTn*3* structure in the 1951017-13 and 1952417-15 strains; an IncFII(K9) fragment (85 kb) was detected as carrying the *mcr-10.1* gene located on the *xerC*-*mcr-10.1*-IS*Ec36* structure in the 2022KU-00264 strain; an IncHI1B/FIB plasmid contig (>200 kb) was found to be harboring the *bla*_NDM-1_ gene, flanked by intact and truncated IS*Aba125* elements in the BWH-P-GER-6 strain; an IncA plasmid contig (>200 kb) was detected as housing the *bla*_KPC-4_ gene encoded within a truncated Tn*4401* element in the BWH-P-GER-1 strain; and IncF plasmid contigs (>100 kb) were identified as harboring the *bla*_KPC-2_ gene with Tn*4401a* and Tn*4401b* isoforms in the 1613525 and 2022DK-00156 strains, respectively.

**Figure 2:**
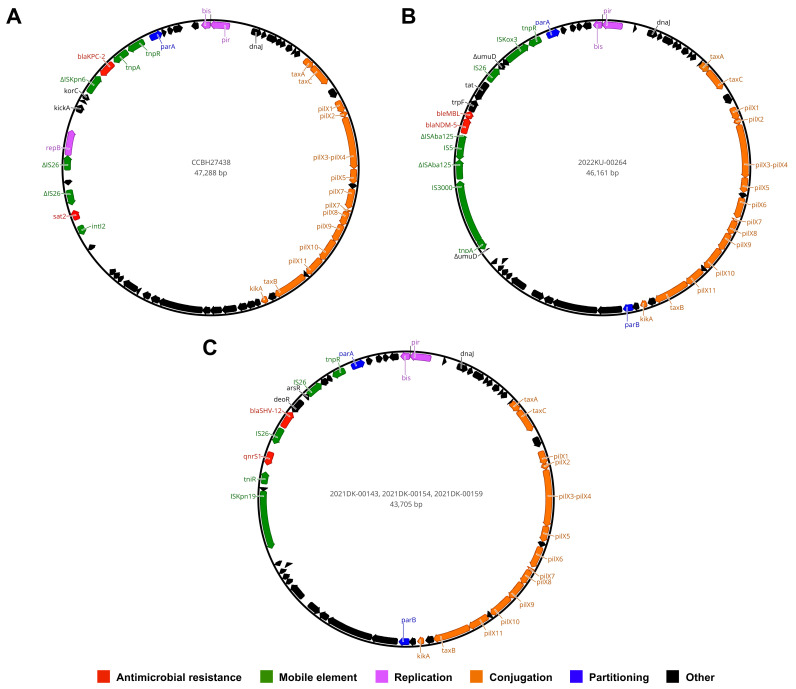
Circular maps of IncX3 plasmids harboring clinically relevant ARGs in *P. gergoviae* strains. (**A**) IncX3-IncU plasmid carrying the *bla*_KPC-2_ gene in CCBH27438 strain. (**B**) IncX3 plasmid housing the *bla*_NDM-5_ gene in 2022KU-00264 strain. (**C**) IncX3 plasmid carrying *bla*_SHV-12_ and *qnrS1* genes in 2021DK-00143, 2021DK-00154, and 2021DK-00159 strains.

In-depth genomic analysis revealed complete sequences of IncX3 plasmids ranging from 43 to 47 kb in length, carrying genes related mainly to antimicrobial resistance, mobile elements, replication, conjugation, and partitioning. An IncX3-IncU plasmid harboring the *bla*_KPC-2_ gene on a non-Tn*4401* (NTE_KPC_-Ic) was identified in CCBH27438 (Figure 2A), while an IncX3 plasmid housing the *bla*_NDM-5_ gene located on the Δ*umuD* genetic structure containing IS*3000*, ΔIS*Aba125*, IS*5*, *ble*_MBL_, *trpF*, *tat*, and IS*26* (Figure 2B) was detected in the 2022KU-00264 strain. IncX3 plasmids from the 2021DK-00143, 2021DK-00154, and 2021DK-00159 strains harbored the *bla*_SHV-12_ gene flanked by IS*26* and the *qnrS1* gene located proximally to *tniR* and IS*Kpn19* (Figure 2C). The presence of the aforementioned ARGs and Inc groups in different plasmid contigs was also detected in other strains, supporting the conclusion that these same plasmids may also be circulating among *P. gergoviae* strains. BLASTn analysis showed that partial or complete plasmid sequences presented high query coverage (>99%) and nucleotide identity (>99%), with other sequences from *Enterobacteriaceae* (e.g., *Salmonella enterica*, *Klebsiella pneumoniae*, *Enterobacter* spp., and *Citrobacter freundii*) distributed worldwide.

### 3.6. Virulence Genotyping

Virulence genes that encode a heat shock survival AAA family ATPase (*clpK1*), a lipoprotein NlpI precursor (*nlpI*), an outer-membrane protein complement resistance (*traT*), and a tellurium ion resistance protein (*terC*) were identified among the *P. gergoviae* genomes. Most strains presented only one of the virulence genes found; however, some of them harbored two genes. Strain C3 from Australia was isolated from a patient with cystic fibrosis and presented *clpK1* and *terC* genes, while environmental strains from the United Kingdom carried *terC* and *traT* genes (Figure 1). 

## 4. Discussion

In this study, we present a first comparative genomic analysis of *P. gergoviae* distributed worldwide. Overall, *P. gergoviae* strains were recovered from humans, animals, foods, and the environment, but MDR strains were isolated only from humans and the environment, which may be related to the limited number of food and animal strains analyzed. In this context, it can be said that this species has been circulating at the One Health interface and that MDR strains may also emerge within it. Therefore, AMR is a multifaceted challenge that requires coordinated global activities to reduce the spread of MDR bacteria [17]. Following the emergence of *P. gergoviae* strains as MDR, high-resolution molecular typing methods, including multilocus sequence typing, should be encouraged in order to improve population structure analysis, strain differentiation, international comparisons, and longitudinal studies.

Species belonging to the *Enterobacteriaceae* family, including *E. coli* and *K. pneumoniae*, are known to be potentially pathogenic and MDR [18]. In general, AMR studies are focused on them, while in other species, mainly newer ones, little is known about the AMR’s genetic background. Recently, *Raoultella* species have gained notoriety for being MDR and shedding clinically important ARGs [19,20]. In this regard, *P. gergoviae* emerges as a new species with these traits since it harbors an arsenal of plasmid-mediated ARGs. As enterobacteria are known for their ability to exchange genetic materials [21], *P. gergoviae* also demonstrates the potential to act as a disseminator of ARG-bearing plasmids. Furthermore, *P. gergoviae* strains also carried biocide resistance genes, contributing to the co-selection of AMR and their persistence in clinical settings [22]. 

Among known AMR-related plasmid families in *Enterobacteriaceae*, IncA, IncF, IncH, and IncN stand out for harboring various ARGs. These conjugative plasmids have been reported especially in medically important bacteria [23], and now also in *P. gergoviae*. Curiously, the first report of AMR-related plasmids in *P. gergoviae* was published in the United States in 2022. In this report, large IncA and IncHI1B/FIB plasmids carrying *bla*_KPC-4_ and *bla*_NDM-1_, respectively, were identified as referring to BWH-P-GER-1 and BWH-P-GER-6 strains [24]. Furthermore, IncX3 plasmids have great conjugation ability and high stability, and have been identified as carrying ESBL- or carbapenemase-encoding genes in *P. gergoviae* strains, reinforcing the importance of *P. gergoviae* in the dissemination of ARGs within *Enterobacteriaceae* [25].

Recently, single- and multireplicon plasmids carrying *mcr-9* or *mcr-10* have been identified in different sources. Consequently, *mcr*-positive and carbapenem-resistant *Enterobacteriaceae* strains have emerged worldwide [26,27] and were also described in this study. In addition, the co-occurrence of ESBL- and carbapenemase-encoding genes in *P. gergoviae* strains has been detected, demonstrating a similar genotype to other medically important enterobacteria [28]. Based on the *P. gergoviae* resistome, it is possible to predict antimicrobial susceptibility and highlight the multidrug resistance phenotype. Although some *mcr* genes do not confer resistance to polymyxins in certain bacterial species [29], good correlation between the genotypic and phenotypic resistance for other antimicrobials was documented [30], including in clinical *P. gergoviae* strains [24]. Furthermore, antimicrobial susceptibility may vary substantially from predictions and may even be underestimated since mutational mechanisms were not investigated in this study, suggesting that MDR strains could still be classified as extensively drug-resistant or pandrug-resistant.

Previous studies have reported that *P. gergoviae* causes opportunistic infections and even nosocomial outbreaks [11,31,32]; however, virulence genotyping strengthened that *P. gergoviae* is a low-virulence bacterium [24], although virulence databases are still limited in the case of this species. Indeed, the role of virulence genes in the pathogenicity of this species needs to be further investigated. Worryingly, *P. gergoviae*-derived infections, especially those caused by carbapenem-resistant strains, can be difficult to treat, representing a significant healthcare challenge and serious public health concern [33]. As described in high-risk clones of *E. coli* and *K. pneumoniae* [34], the convergence of virulence and AMR did not occur in *P. gergoviae.* In addition, no species- and host-specific genes related to AMR or virulence were identified, evidencing an acquired and diverse genetic background.

## 5. Conclusions

In summary, this study provides the first genomic overview of *P. gergoviae* strains circulating at the One Health interface. Comparative analysis revealed a highly diverse population that harbored an arsenal of ARGs. Conjugative plasmids were identified as carrying clinically important ARGs embedded in conserved genetic contexts, evidencing their capture of other medically important bacteria. In this sense, *P. gergoviae* is emerging as a new multidrug-resistant species belonging to the *Enterobacteriaceae* family. Therefore, more studies investigating the genetic background of AMR in non-clinically relevant enterobacteria should be encouraged and continuous epidemiological genomic surveillance of *P. gergoviae* should be required. 

## Figures and Tables

**Table 1:** WGS-predicted phenotype based on plasmid-mediated ARGs of *P. gergoviae* genomes.

Strain ^1^	Antimicrobial Agent ^2,3^																			Pattern ^4^
	COL	AMP	AMC	PTZ	CFO	CAZ	CPM	ATM	MPM	CIP	STP	GEN	AMI	TET	MIN	TGC	SXT	CHL	FOS	
1613625 *	S	R	R	R	R	R	R	R	R	S	S	S	S	S	S	S	S	S	S	MDR
2021DK-00143 *	S	R	R	R	R	R	R	R	R	R	R	R	R	R	S	S	R	R	S	MDR
2021DK-00148 *	S	R	R	R	R	R	R	R	R	R	S	S	S	S	S	S	R	R	S	MDR
2021DK-00170 *	R	R	R	R	R	R	R	R	R	S	R	R	S	R	S	S	R	R	S	MDR
2022DK-00148 *	R	R	R	R	R	R	R	R	R	S	R	S	S	S	S	S	S	R	S	MDR
2022DK-00154 *	S	R	R	R	R	R	R	R	R	R	R	R	R	R	S	S	R	R	S	MDR
2022DK-00156 *	R	R	R	R	R	R	R	R	R	S	R	R	S	S	S	S	R	R	S	MDR
2022DK-00159 *	S	R	R	R	R	R	R	R	R	R	R	R	R	R	S	S	R	R	S	MDR
2022KU-00264 *	R	R	R	R	R	R	R	R	R	R	S	R	S	S	S	S	S	S	S	MDR
2023BM-00012 *	S	S	S	S	S	S	S	S	S	S	S	S	S	S	S	S	S	S	S	–
2023BM-00013 *	S	S	S	S	S	S	S	S	S	S	S	S	S	S	S	S	S	S	S	–
214632 *	R	R	R	R	R	R	R	R	R	R	R	R	R	R	S	S	R	S	S	MDR
40874 *	S	S	S	S	S	S	S	S	S	S	S	S	S	S	S	S	S	S	S	–
AK5227 *	S	R	R	R	R	R	R	R	R	R	R	R	R	R	R	S	R	S	S	MDR
ATCC 33028 *	S	R	S	S	S	S	S	S	S	S	R	R	S	R	S	S	R	R	S	MDR
BWH-P-GER-1 *	S	R	R	R	R	R	R	R	R	R	R	R	R	R	S	S	R	R	S	MDR
BWH-P-GER-6 *	S	R	R	R	R	R	R	R	R	R	R	R	R	R	S	S	R	R	S	MDR
C3 *	S	S	S	S	S	S	S	S	S	S	S	S	S	S	S	S	S	S	S	–
C7B *	S	S	S	S	S	S	S	S	S	S	S	S	S	S	S	S	S	S	S	–
CCBH27438 *	S	R	R	R	R	R	R	R	R	R	R	S	R	S	S	S	S	S	S	MDR
DL84A27 *	S	S	S	S	S	S	S	S	S	S	S	S	S	S	S	S	S	S	S	–
DS82E24 *	S	S	S	S	S	S	S	S	S	S	S	S	S	S	S	S	S	S	S	–
DTU-2020-1001845-1 *	S	S	S	S	S	S	S	S	S	S	S	S	S	S	S	S	S	S	S	–
DTU-2021-1003198-1 *	S	S	S	S	S	S	S	S	S	S	S	S	S	S	S	S	S	S	S	–
ECO77-3 *	S	S	S	S	S	S	S	S	S	S	S	S	S	S	S	S	S	S	S	–
FDAARGOS-386 *	S	S	S	S	S	S	S	S	S	S	S	S	S	S	S	S	S	S	S	–
JB83E35 *	S	S	S	S	S	S	S	S	S	S	S	S	S	S	S	S	S	S	S	–
JP84E9 *	S	S	S	S	S	S	S	S	S	S	S	S	S	S	S	S	S	S	S	–
JS81F13 *	S	S	S	S	S	S	S	S	S	S	S	S	S	S	S	S	S	S	S	–
MGH173 *	R	R	R	R	R	R	R	R	R	R	R	S	S	R	S	S	R	R	S	MDR
MGH183 *	R	R	R	R	R	R	R	R	R	R	R	S	R	R	S	S	R	S	S	MDR
Microbial *	R	R	R	R	R	R	R	R	R	S	R	S	R	R	S	S	R	R	S	MDR
SF84F43 *	S	S	S	S	S	S	S	S	S	S	S	S	S	S	S	S	S	S	S	–
SN86A38 *	S	S	S	S	S	S	S	S	S	S	S	S	S	S	S	S	S	S	S	–
SS84A28 *	S	S	S	S	S	S	S	S	S	S	S	S	S	S	S	S	S	S	S	–
PG1351 ^^^	S	S	S	S	S	S	S	S	S	S	S	S	S	S	S	S	S	S	S	–
FB2 ^$^	S	S	S	S	S	S	S	S	S	S	S	S	S	S	S	S	S	S	S	–
H11 ^$^	S	S	S	S	S	S	S	S	S	S	S	S	S	S	S	S	S	S	S	–
1951017-11 ^#^	R	R	R	R	R	R	R	R	R	R	R	R	R	R	S	S	R	S	S	MDR
1951017-12 ^#^	R	R	R	R	R	R	R	R	R	R	R	R	R	R	S	S	R	S	S	MDR
1951017-13 ^#^	R	R	R	R	R	R	R	R	R	R	R	R	R	R	S	S	R	S	S	MDR
1951017-14 ^#^	R	R	R	R	R	R	R	R	R	R	R	R	R	R	S	S	R	S	S	MDR
1951017-15 ^#^	R	R	R	R	R	R	R	R	R	R	R	R	R	R	S	S	R	S	S	MDR
1952417-11 ^#^	R	R	R	R	R	R	R	R	R	R	R	R	R	R	S	S	R	S	S	MDR
1952417-12 ^#^	R	R	R	R	R	R	R	R	R	R	R	R	R	S	S	S	R	S	S	MDR
1952417-13 ^#^	R	R	R	R	R	R	R	R	R	R	R	R	R	R	S	S	R	S	S	MDR
1952417-14 ^#^	R	R	R	R	R	R	R	R	R	R	R	R	R	R	S	S	R	S	S	MDR
1952417-15 ^#^	R	R	R	R	R	R	R	R	R	R	R	R	R	R	S	S	R	S	S	MDR

^1^ *, strains from humans; ^^^, strain from dog; ^$^, strains from foods; ^#^, strains from the environment. ^2^ S in green color, susceptible; R in red color, resistant. ^3^ Antimicrobial categories as follows: polymyxins (COL, colistin), penicillins (AMP, ampicillin), penicillins + β-lactamase inhibitors (AMC, amoxicillin-clavulanic acid), antipseudomal penicillins + β-lactamase inhibitors (PTZ, piperacillin-tazobactam), cephamycins (CFO, cefoxitin), extended-spectrum cephalosporins; 3rd and 4th generation cephalosporins (CAZ, ceftazidime; CPM, cefepime); monobactams (ATM, aztreonam); carbapenems (MPM, meropenem); fluoroquinolones (CIP, ciprofloxacin); aminoglycosides (STP, streptomycin; GEN, gentamicin; AMI, amikacin); tetracyclines (TET, tetracycline; MIN, minocycline); glycylcyclines (TGC, tigecycline); folate pathway antagonists (SXT, trimethoprim-sulfamethoxazole); phenicols (CHL, chloramphenicol); and phosphonic acids (FOS, fosfomycin). ^4^ Classification as MDR according to Magiorakos et al. [9]; MDR, multidrug-resistant; –, non-MDR.

## Data Availability

Data are contained within the article.
